# Carob: A Mediterranean Resource for the Future

**DOI:** 10.3390/plants13091188

**Published:** 2024-04-25

**Authors:** Maria Amélia Martins-Loução, Pedro José Correia, Anabela Romano

**Affiliations:** 1 cE3c—Center for Ecology, Evolution and Environmental Change & CHANGE—Global Change and Sustainability Institute, Faculdade de Ciências, Universidade de Lisboa, 1749-016 Lisboa, Portugal; 2 MED—Mediterranean Institute for Agriculture, Environment and Development & CHANGE—Global Change and Sustainability Institute, Faculdade de Ciências e Tecnologia, Universidade do Algarve, Campus de Gambelas, Ed. 8, 8005-139 Faro, Portugal; pcorreia@ualg.pt

**Keywords:** carob, *Ceratonia siliqua*, Mediterranean, climate change, industrial applications, afforestation, environmental benefits, production and cultivation

## Abstract

For centuries, the carob tree (*Ceratonia siliqua* L.) has contributed to the economy of the Mediterranean basin, mainly as food for livestock. Nowadays, the value of the carob tree extends far beyond its traditional uses, encompassing a wide range of industries and applications that take advantage of its unique properties and nutritional benefits. Despite its high industrial demand and European indications, there has been a 65% reduction in the area cultivated throughout the Mediterranean area in the 21st century. Given the threats posed by climate change, including reduced water availability and nutrient-depleted soils, there is a growing need to focus on this crop, which is well placed to cope with unpredictable weather. In this review, we use a bibliographic search approach to emphasise the prioritisation of research needs for effective carob tree exploitation. We found enormous gaps in the scientific knowledge of this under-utilised crop species with fruit pulp and seeds of high industrial value. Insufficient understanding of the biology of the species, as well as inadequate agronomic practices, compromise the quantity and the quality of fruits available to the industry. In addition to industrial applications, carob can also be used in reforestation or restoration programmes, providing a valuable crop while promoting biodiversity conservation and soil restoration. The carbon sequestration potential of the trees should be taken into account as a promising alternative in fighting climate change. This bibliographic search has highlighted clusters with different knowledge gaps that require further research and investment. The carob tree has untapped potential for innovation, economic development, and environmental sustainability.

## 1. Introduction

The carob tree (*Ceratonia siliqua* L.) is a dioecious tree belonging to the Fabaceae family, subfamily *Caesalpinoideae*. It is a hardy arboreal plant traditionally cultivated throughout the Mediterranean basin and is also a conspicuous component of the Mediterranean vegetation, forming part of the maquis or garrigue as a shrubby, sclerophyllous plant that is well adapted to drought stress [1,2,3].

For centuries, the carob tree has been a significant contributor to the economy of the Mediterranean basin, primarily as a source of food for livestock [4]. The Egyptians used its fruit to make wine, while the dried fruit pods were used to dye leather, and the seeds were used to make rosaries and decorative necklaces. Although it has long been associated with poverty and hardship, being used for food when there was nothing else to eat or as a commodity, it has remained an important part of the region’s economy [5]. In times of war, both Cretans and Spaniards have experienced these traumatic memories. The carob tree, which is referenced in the Bible as a means of survival for St. John the Baptist in the desert, has become a symbol of safety and resilience in Mediterranean culture. Its pods require minimal care, making it a reliable source of sustenance.

The pods of the carob tree have historically been used as animal feed, providing essential nutrients to livestock in times of scarcity. By providing energy, protein and fibre, carob plays an important role in supporting animal health and productivity while promoting resilience in farming systems and contributing to agricultural sustainability [6]. Carob pods are a rich source of carbohydrates, providing animals with a readily available source of energy to fuel their daily activities and metabolic processes. This energy boost is particularly beneficial for livestock during periods of high activity, growth, reproduction, or lactation. In addition, carob pods contain significant amounts of protein, which contributes to muscle development, tissue repair, and overall animal health. When included in a balanced diet, carob can help meet the protein requirements of livestock, supporting their growth and productivity. Additionally, carob pods are high in fibre, which plays an important role in maintaining digestive health. This is particularly important for ruminants such as cattle, sheep, and goats, whose digestive systems rely on fibre fermentation for optimal function.

Over the last half century, carob cultivation regained industrial processing importance after a period of decline [7,8,9]. Extensive scientific research on agronomy and cultivation was initiated [10,11,12] and the nutritional benefits of the seeds were discovered [13].

Currently, the carob tree holds value beyond its traditional uses, with a wide range of industries and applications taking advantage of its unique properties and nutritional benefits. Carob is a versatile ingredient with applications in various industries, including in food and beverages, pharmaceuticals, cosmetics, animal feed production, and agroforestry systems. It offers natural and sustainable solutions to a range of industrial processes [13,14]. Its fruit, the carob pod, is the primary source of its industrial value. To utilize the pod, the first step is to separate the seed from the pulp through a process called de-seeding or kibbling.

The seed is composed of germ, endosperm, and husk. Grinding the endosperm produces the food additive LBG (E-410), also known as locust bean gum. This additive is widely used as a thickener and stabiliser in various food and non-food products. The economic importance of the carob pod is justified by its non-toxic properties and high degree of purity, which make it suitable for industrial use [13,15]. LBG is a versatile ingredient in the food industry, valued for its excellent thickening and gelling properties. It is commonly used to stabilise and texturise products such as sauces, dressings, ice cream and dairy desserts. LBG is a popular choice for manufacturers seeking natural, plant-based alternatives to synthetic thickeners and stabilisers. Additionally, LBG finds use in various industrial applications, including papermaking, textile printing, and cosmetics. The hydrocolloids in carob seeds make it suitable for use in adhesives, coatings, and cosmetic formulations such as creams, lotions, and hair-care products. Moreover, the germ of the seed contains about 20% protein-rich food flour, which displays a high content of vitamins B1 and B2 and is used in baby food. The seed embryo, or germ flour, contains 50% caroubin, a water-insoluble protein with similar rheological properties to gluten [16]. Due to its low level of cysteine, caroubin can be a suitable gluten substitute [16,17].

The pulp, considered a by-product of the carob-pod industry, is an important source of nutrients, including carbohydrates, minerals, amino acids, and vitamins [18,19]. It is used in various of culinary applications to enhance flavour, texture, and nutritional value. Furthermore, the carob pulp shares comparable nutritional, functional, and organoleptic characteristics with cocoa. It has the added benefit of being food that is free from theobromine and caffeine and has a low-fat content [20]. The pulp is a natural sweetener and a healthier alternative to refined sugar. Unlike sugar, carob is low in calories and does not cause a rapid increase in blood sugar levels, making it an excellent option for people with diabetes or those seeking to maintain stable energy levels. The pod has become a novel functional food with multiple uses in the pharmaceutical and nutraceutical industry due to scientific research investment in the chemistry of the whole fruit [21,22,23,24].

The carob tree has also long been recognised for its potential health benefits and has been used for centuries in traditional medicine to treat a range of health problems, including diarrhoea, diabetes, and hypertension. Its pods, seeds, and leaves have been found to offer health-promoting properties [20,23,25,26,27]. Carob has been reported to possess various several pharmacological activities, such as antioxidative, anti-diarrhoea, antibacterial, anti-ulcer, anti-inflammatory, and anti-diabetic effects [14]. The pulp is rich in dietary fibre, which plays an important role in digestive health. Fibre regulates bowel movements, prevents constipation, and supports the growth of beneficial gut bacteria. Carob pulp may contribute to overall gastrointestinal well-being by promoting regularity and aiding digestion.

Given the challenges posed by climate change in the Mediterranean basin, it is necessary to explore new approaches to sustainably cultivate carob tree agroecosystems under rainfed conditions. The loss of biodiversity is a pressing issue, and the FAO is actively promoting the conservation and use of farmers’ varieties/landraces in the region [28]. The promotion and conservation of native Mediterranean fruit tree species that are adapted to global climate change and that are particularly suitable for soil conservation is crucial. One such species is the carob tree, which holds significant economic importance for the Iberian Peninsula, accounting for 41% of total carob production in the Mediterranean basin [9]. Carob is considered an under-utilised fruit tree species that is cultivated in the Mediterranean basin countries of Europe and is recommended for conservation under the frame of the European programme for the conservation and utilisation of germplasm (EC regulation N° 1467/94) [29].

Despite its high industrial demand and European regulations, there has been a reduction of 65% in the area cultivated throughout the Mediterranean area in the 21st century [9]. Given the threats of climate change, particularly the loss of water availability and poor-nutrient soils, it is increasingly important to focus on this crop, which is well suited to facing unpredictable weather events [30,31,32,33]. After over 40 years of research in the Mediterranean region, we conducted a bibliographic search to identify research priorities for effective carob tree exploitation. Here, we conduct an advanced search analysis based on our bibliographic knowledge of the carob tree to identify the most significant scientific knowledge gaps that require further investigation. This review aims to identify knowledge clusters or gaps that can enhance the efficiency of gathering funders or policy makers to promote this crop in the Mediterranean basin. The promotion of this crop can be a sustainable and prospective option for the region, taking into account environmental, social, and economic factors.

## 2. Results and Discussion

### 2.1. Searches and Screening

From conducting refined searches on the Scopus database, it is noteworthy that there has been a significant increase in publications on carob in the past 20 years (Table 1). The majority of publications focus on the industrial applications of carob, including its health benefits and nutritional value for human consumption (54% of the total).

All the references before the 1980s, including two others from the 19th century, describe the plant and highlight the importance of its fruit for feed and food. They also discuss its potential as a tree crop in various parts of the world [30,34,35,36,37,38,39,40,41]. These previous works were important for understanding carob biology and ecology as well as its potential applications. They accounted for 40% and 24% of the total number of publications during this period (Table 1). At the end of the 1970s, scientific research on carob began to take off. The plant was presented as a model of resilience in Mediterranean conditions and as a potential crop with several productive varieties. Approximately 10% of the total number of publications (Table 1) focused on this topic, particularly in rustic areas and arid climates [42,43,44,45,46].

From 1980 to 2000, 40% of the scientific studies focused on describing mechanisms of the carob plant’s drought resistance [3,47,48,49], and its ecophysiological responses under different nutrient conditions, both in the laboratory and in field plantation settings [50,51,52,53,54,55,56,57]. The research aimed to improve the ecological understanding of this plant, which is known to be easily cultivated by rural communities. At that point, specialised farming was not a priority because farmers did not consider carob to be an economically valuable crop but rather a traditional Mediterranean tree with a small but continuous profit.

During this period, other scientific works on health focused on using carob-pod sugars as an alternative to sugar cane [58,59,60], Additionally, research was conducted on the production of gum from the seed endosperm and its interaction with other biological gums [61,62,63,64]. The aim was to develop an improved industrial process for extracting sugar from the pods and an efficient process for utilizing the gum present in the seed endosperm.

Despite the gaps in justifying investment in a truly economic crop, the scientific developments of this century have been significant. Much work has been conducted this century on its applications in key areas of health and the circular economy (37% in the last 15 years). This innovative work has highlighted the potential of this neglected crop. Unfortunately, this work has not been supported by agricultural production technologies, which have made it impossible to guarantee the quantity and quality of the industrial product. In some areas, such as in the varietal characterisation and environmental responses, approaches have been repeated over time, depending on the scientists’ research focus, available opportunities in each country, or the outcome of international projects.

### 2.2. Carob Origin and Present Varieties

The carob tree has been present in the eastern Mediterranean since before the advent of its agriculture. This information is based on archaeobotanical finds from over 43,000 years ago (BC), discovered in what is now Israel [65]. For years, its centre of origin was considered unclear, with some believing that the carob tree did not originate in the Mediterranean but rather in an eastern region, either in Oman, southeast of the Arabian Peninsula, or near the African horn, north of Somalia. Recent research suggests that the carob tree is native to the eastern Mediterranean region. This hypothesis is supported by three pieces of evidence. Firstly, the discovery of another species of the *Ceratonia* genus—*C. oreothauma*—in Yemen. Secondly, pollen and macro-remain records of *C. siliqua* in the east. And thirdly, the scarcity of local names attributed to the carob tree in the west [66]. Zohary [67] suggests that the carob tree, along with the olive tree, laurel, myrtle, and dwarf palm [68], is a relic of an Indo-Malay floral species of xerotropical origin. The carob tree was believed to have been domesticated in the east.

Viruel et al. [69] identified the carob tree as of pre-Mediterranean lineage with ancestors distributed around the Tethys Sea. The study estimated an origin near the Oligocene/Miocene, which is linked to the tropical–subtropical paleo-Mediterranean Sea, as previously stated by Zohary [65]. However, the presence of four lineages of *Ceratonia siliqua* depicted through the phylogeography study makes this difficult to reconcile with the proposed eastern domestication hypothesis [69]. These lineages evolved before the major civilisations of the Fertile Crescent and the Mediterranean, supporting the persistence of the carob tree in Moroccan and Iberian refugia.

The greatest evolutionary forces on this species have been selection and expansion by Mediterranean man, as well as their ecological adaptation to environmental conditions. For example, in arid conditions, as opposed to sub-humid to semi-arid conditions, trees show greater variability within the same cultivar. This variability can be greater than the variability between populations. However, phylogenetic and genetic studies indicate that the carob has low levels of allelic richness [69,70,71]. This is consistent with vegetative propagation in the western Mediterranean and a significant decline during the last interglacial period. Carob is a neglected species and is particularly vulnerable to genetic erosion due to the lack of rational nursery activity and genetic improvement programs.

Carob trees are widespread in Mediterranean region and have been extensively studied by researchers from various countries. The most important parts of the plant, namely the pod and the seeds, have been the subject of studies by Kalaitzakis, Battle, Garnit, Biner, and Haddarah, among others [4,21,72,73,74]. Several studies have also investigated other parts of the plant, such as flowers in Portugal [75,76] and leaves [77] and bark in Morocco [78]. However, these studies only used morphological or chemical characteristics to evaluate cultivars. Molecular approaches have recently been used to complement genotypic variability studies from Lebanon to the Iberian Peninsula [70,71,79,80,81,82,83,84,85]. The genetic resources of Mediterranean carob trees are limited in number and exhibit high genetic variation in various traits, including pod size, shape and colour of the pod, and seed yield.

Although there have been scientific advances in characterising cultivars throughout the Mediterranean basin and in carob phylogeography, there is still a gap in knowledge regarding the best cultivars for each microclimate niche, soil characteristic, and geography. The main selection objectives have traditionally been large pod size, high pulp content, and high sugar content. It is well known that there is a negative correlation between pulp and seed content. In the early days, farmers selected varieties rich in pulp for animal feed. However, technological and industrial developments in the last two decades of the twentieth century have led farmers to place greater value on varieties that produce large quantities of seed for the commercial value of the gum.

Carob is described as a dioecious plant in the wild [66], but hermaphrodite cultivars are also present [45], particularly in the eastern coastal belt of Spain [4]. It appears that hermaphroditism evolved from separate sexes without any evolutionary cost [83]. The existence of these rare varieties in Spain is not explained or justified. The environmental isolation of the Balearic Islands probably favours these rare varieties [8]. On the other hand, if carob plants in the wild have separate sexes, a precise mechanism must be present to determine sex. This mechanism may not necessarily be connected to the genes but could be influenced by environmental conditions [86]. The exact mechanism that carob uses to determine sex is still an open question that requires further investigation.

Global climate change is a known threat to some dioecious plant species. Higher temperatures favour male plants [87], which could result in a range expansion of carob into the cooler northern climates. This expansion could lead to changes in their spatial abundance and exploitation. Carob has different male and female cultivars [88], but under farm conditions, its production relies on grafted plants. The quantity of male plants dispersed on farms is dependent on the cultivar, as well as on regional and cultural practices. This knowledge is based on empirical evidence rather than scientific knowledge. Selecting the appropriate variety for a specific pedo-climatic environment is one of the most important issues in carob exploitation. Therefore, it is necessary to explore the genetic material in each producing country. In 2013, a germplasm collection consisting of 44 accessions was established in Portugal, which is now being monitored annually to provide the necessary vegetative material for grafting. Furthermore, in Spain, there is a germplasm collection bank that is located in three different areas [4].

Genetic improvement of carob cultivars has so far been carried out only empirically. The lack of a planned carob breeding programme may be due to its relatively minor agronomical importance. Therefore, research into the genetic material in each producer country is necessary. Despite the efforts made, the bibliography on cultivated varieties represents only 14% of the total references for the last 20 years (Table 1). Scientific understanding of the expression of sexuality and pollination mechanisms, as well as the impact of environmental conditions on them, will be necessary for future breeding programmes. This highlights the limited knowledge available on the genetic resource characteristics and environmental responses of the carob. Developing best management practices for carob orchards to maximise carob production will require a better understanding of these factors.

### 2.3. Carob Production

Water and nitrogen availability are the two most important factors for plant establishment and growth. The rainfall amount and distribution throughout the year are critical for water availability, and this is dependent on the climate. In Mediterranean climates, rainfall distribution over the months is more critical than the amount. In terms of nutrient availability, nitrogen is the primary element that limits production [89], along with water availability [57]. In these environments, plant roots cannot efficiently absorb the required amounts of nitrogen and withstand drought stress. To facilitate this process, associations with mycorrhizal fungi [90,91,92,93] and soil bacteria [94] can be established. Carob, like most *Caesalpinoideae*, is not a nitrogen-fixing plant [10,95,96], although some rhizobacteria have been found near carob roots [97]. Carob is highly adaptable to different environmental conditions, as evidenced by its ability to adjust its leaf area, number of branches, and root branching [98,99,100].

The carob tree grows mainly from seed under conditions that are stable and well characterized [101,102]. Although vegetative propagation from cuttings has been reported for certain Spanish cultivars [103], this method has not been as successful in achieving high yields as it has been for Portuguese cultivars [11]. The conditions most suitable for rooting [104,105] and acclimatisation [106] have also been evaluated. However, due to the ease of the germination process [101,102] and the high viability of the seeds, this process is much more accessible. Proper preparation of plants in the nursery is crucial for successful planting and survival, especially when grafting is involved [8,107]. Grafting the rootstocks before field transplantation is a practice that may lead to early bearing, thus reducing the initial unproductive period [107]. Currently, grafted plants are very expensive, and the grafting procedure may not always be successful at the nursery due to several variables. To reduce the initial cost of the investment, farmers are using rootstocks obtained from seeds. However, this may delay the initial fruit production, which has economic significance. Carob, like most tree crops, matures slowly. Most cultivars do not begin commercial production until 6–7 years after planting [108,109].

Carob is a sustainable crop tree that offers a wide variety of economically profitable derived products and ensures stable prices. It requires less water and intensive care compared with other crops [56,110]. However, it is not frost tolerant [111]. Most evergreen Mediterranean, including the carob tree, have hard, leathery leaves that help control water loss [2,3]. Additionally, the carob tree has evolved the ability to efficiently extract water from the soil to compensate for atmospheric losses. This is facilitated by its deep root system and rapid changes in its water potential [112,113]. As a species capable of surviving and producing with only 250 mm of rainfall, it can be inferred that this species has specific mechanisms for adapting to water stress.

Fruit production per tree is significantly lower in soils with low organic matter [114]. As a result, carob is primarily being used as a forestry species with minimal input and low income for farmers. These characteristics have restricted the crop’s usage, and predicting yield, particularly after the late summer harvest, is challenging. Supplementary drip irrigation during the initial years of planting can enhance the diversification and revitalisation of coastal agricultural areas with an arid Mediterranean climate [8]. Additionally, the input of nutrients, such as mineral fertilizers and/or organic matter, can increase fruit production and promote vegetative growth [110,115]. New plantations are now being irrigated not only in the first years after planting but also in subsequent growing seasons, from early spring until late summer. However, the amounts of irrigation water vary greatly even under the same pedo-climatic conditions. For similar tree ages and with 208 trees per hectare, values may range from 600 to 2000 m^3^ per hectare. Irrigation amounts are often adjusted by farmers without considering scientific recommendations, despite the fact that higher amounts should be established based on the evapotranspiration of the site and the canopy cover. Typically, a farmer’s decision is based on the water resources available for the growing season. The influence of climate on carob-tree yield is complex, and several factors limit the success of flowering, pollination, and fruit set, leading to irregular yields between years [110,116]. Ultimately, this leads to an inefficient and irregular profit for carob orchards.

With the current scenario of global warming, changes in temperature and precipitation can cause yield fluctuations in dry-farming orchards. A study conducted in Portugal over 30 years found no relation between yield and temperature. However, it did find that precipitation during the hydrological year had a negative impact on yield, particularly with autumn rainfall [109]. Similarly, Sidina et al. [117] found correlations between morphological characters, geographic parameters, and precipitation in Morocco. Correlation analysis revealed that latitude had a positive effect on pod characteristics, whereas altitude had a negative effect. These factors were found to impact the quality of fruits and their seed contents. Furthermore, in an aridity gradient, pod-related traits showed greater variability than seed traits [100]. The results suggest that there is still a need to investigate the direct impact of climatic variables and geographic conditions on carob production.

The proportion of published studies on the agronomic aspects of carob production (16%; Table 1) is similar to that on the ecology of carob (14% in the last 20 years, compared with 40% in the previous period) (Table 1). However, this has not contributed to filling the knowledge gap regarding the direct impact of climatic variables and geographical conditions on carob production. The experimental conditions are different, and extrapolation from laboratory to field is not feasible. The variable fruit production creates a disadvantage compared with other industrial crops, resulting in regional disparities in competitiveness. The climate can lead to a highly variable fruit production, ultimately resulting in inefficient and irregular profits for carob orchards.

### 2.4. Industrial Applications

The conservation of germplasm for under-utilised fruit tree species grown in the Mediterranean-basin countries of Europe has received some attention. Accessions of carob are held both in situ and ex situ by 11 institutions in France, Greece, Italy, and Spain [29].

In recent decades, the carob tree has been the subject of numerous publications, primarily due to its technological and biotechnological applications (54% of the total). These applications have direct relevance to the pharmaceutical and food industries, as well as to the production of bioethanol. Among these technological publications, 35% are patent registrations. Food industry applications are mainly related to LBG and its purity characteristics [118,119,120]. The bibliographic evaluation indicates that this crop remains of global interest [121]. This is particularly evident since some of the publications or industrial developments have been carried out by researchers outside the Mediterranean basin, who are linked to research units or multinational companies [122]. The economic value of the crop therefore depends on increasing the value of the fruit as a whole and diversifying its uses [123].

Recent publications have focused on the health effects of carob pods [124]. Indeed, carob pods have been reported to exert anti-inflammatory, antimicrobial, anti-diarrhoeal, antioxidative, anti-ulcer, anti-constipation, and absorption-inhibitory activities in the gastrointestinal tract [14]. These health-promoting properties may be attributed to its bioactive compounds, including phenolic acids, flavonoids, and tannins [26]. These compounds have functional properties and are associated with numerous health benefits such as anti-inflammatory, antioxidative, anti-aging, and anti-diabetic effects. Antioxidants can aid in protecting the body against oxidative stress and inflammation, which are underlying factors in many chronic diseases, such as heart disease, cancer, and neurodegenerative disorders [19,24]. By adding carob to their diet, individuals may be able to reduce their risk of developing these conditions and support their overall health and longevity. It is also a good alternative to commercially available high-sugar soluble cocoa powder [125]. However, further research is required to fully understand and harness the medicinal potential of carob. This will open up opportunities for pharmaceutical and nutraceutical applications [126].

### 2.5. Environmental Role

Climate change has a significant impact on human societies and the way they choose to prioritise their strategies for adaptation and mitigation. The strategies addressed are affected by the extent of climate change in each region, which also impacts the level of transformation that societies will experience [127]. The Mediterranean region is highly susceptible to climate change [128]. It is located in a transition zone between mid-latitude and sub-tropical regimes, resulting in a variety of weather types and climate zones [129]. The region has been shaped by long-lasting human-induced habitat changes due to its geological past, geography, and climate. Blondel and Aronson [130] explain that the complex coevolution is responsible for the dynamics of the present biodiversity and agroecosystem diversities in the Mediterranean region.

The Mediterranean region is currently confronted with a variety of potentially catastrophic challenges. These include diversified socioeconomic interests, intensive migration movements, global warming, changes in soil water availability, land-use changes, the spread of invasive species, and changes in traditional agriculture and livestock activities [131,132]. The economic growth recorded in the second half of the 20th century has led to rural abandonment in several regions of the Mediterranean basin, particularly in the Iberian Peninsula. The agricultural policy, aimed at doubling food production by 2050, has resulted in intensive agricultural activities that have caused critical levels of habitat loss and the erosion of genetic variability in traditional Mediterranean crops, such as olive, carob, almond, and fig trees.

In many Mediterranean regions, farmers traditionally intercropped olive, carob, almond, and fig trees to exploit their different fruits. However, olives and almonds were eventually abandoned in favour of an intensified irrigation system. This transformation led to the conversion of mixed carob, almond, and fig orchards into a maquis ecosystem. Over the years, these stable ecosystems have been transformed into “part-time agriculture”, where carob pods are used as a valuable source of income during less productive periods, or into “tourist agroforestry”.

Agroforestry is recognized as a strategy for mitigating the threats posed by climate change, specifically as a means of biological carbon (C) sequestration. In arid and semi-arid Mediterranean climates, farmers may receive payments as revenue from the carbon market, which can cover other management costs. Payments for ecosystem services have been proposed for cork-oak savannah ecosystems in Portugal [133]. These payments have primarily been tested in carob orchards [116]. When implementing dry-farming agricultural strategies for under-utilised fruit tree crops, such as carob, it is important to consider not only crop productivity but also estimates of their potential carbon sequestration as an agroforestry system [134]. This requires combining information on the aboveground carbon stocks and soil carbon values, as in other reforestation situations [135].

Improving production is closely linked to climate constraints. As water scarcity is expected in the short term, irrigation may not be possible for the carob tree. In regions of the Mediterranean basin where irrigated tree crops such as oranges and avocados dominate, carob is not a competitive crop. Therefore, agricultural policies must ensure that this slow-growing species benefits from specific funding and support. In dry-farming systems, mixed orchards can therefore offer the potential for higher incomes while maintaining crop diversification and biodiversity. This is a true agroforestry system [79]. The benefit of carbon sequestration [116] may represent 125–300% of the income, turning CO_2_ equivalents into a novel ecological economic incentive that may provide a new income for farmers while ensuring carob ecosystem services.

Methodological difficulties in estimating the carbon stock of biomass and the extent of soil carbon storage under varying climatic conditions are a hindrance due to the lack of reliable estimates. More studies should be conducted to test different approaches based on different soil characteristics [114]. Unfortunately, our knowledge of these issues is still rudimentary. This explains the paucity of publications on the subject over the last 20 years. Only 1.5% of studies (Table 1) have focused on the benefits of the carob dry-farming system compared with the continuing interest in ecological responses over the same period (14%, Table 1). It is important to note that the environmental benefits of these systems are often overlooked due to the challenges associated with them.

## 3. Materials and Methods

### 3.1. Searches

The publication databases searched were the generic databases Google Scholar, Scopus, and the Document Repository of the Food and Agriculture Organisation (FAO). The search terms used were “carob” and “*Ceratonia*”. Advanced searchers included “health”, “nutrition”, “varieties”, “genetics”, “crop production”, “crop cultivation”, “farming” and “dry-farming”, “environment”, and “agroforestry”. These are the current issues that tend to affect the interests of farmers. The terms “health” and “nutrition” are related to the economic applications of carob, which are important for how farmers and policymakers perceive and evaluate carob as an economic investment. The terms “varieties” and “genetics” are closely linked to the genetic variability of cultivars and, consequently, to pod characteristics and economic value. “Crop production” and “cultivation” are essential for farming investment and can provide cost–benefit profits for farmers that are comparable to other highly valued fruit crops in the market. The terms “farming” and “dry-farming”, “environment”, and “agroforestry” are related to the ecophysiological responses under different environmental conditions. They also address the current threats of increased drought and the potential for economic gain from carbon sequestration and carbon markets. These measures are being taken to adapt to climate change and to protect the environment.

### 3.2. Method of Screening and Data Coding

After conducting a thorough search of the FAO repository, we decided not to use it as it only referred to carob applications as a food resource and did not cover any of our advanced searches. The Journal Citation Reports (JCR) databases from the last two decades were consulted to ensure that there were no missing references to carob in our search. We found a higher number of publications related to “carob” and “*Ceratonia*” on Google Scholar (49,500) compared with Scopus (351). Due to this disparity and for a more focused selection, we used the advanced search words in both databases. The search and eligibility criteria for the databases were checked by randomly screening all publications and comparing the information from Google Scholar and Scopus, together with a re-check in the JCR database. Scopus was chosen as the work database due to its more accurate screening of words, resulting in fewer repetitions and false data coding compared with Google Scholar. We added additional references, including works from before 1980 and even from the 19th century, to the list of 363 bibliographic references. All references have been entered into the EndNote programme.

The searches were conducted in January 2024 and included only English-language works to ensure a strong foundation for international dissemination and peer review. Only a few exceptions were made for dissertations or books written in Portuguese or Spanish. To gain a better understanding of the importance of research studies on the different evaluated aspects, we divided the number of publications into two major time intervals: the first 20 years of this century and the last 20 years of the previous century (Table 1). Upon sorting the articles by the selective searches mentioned above, we realised that many of them covered different topics. Therefore, we included them in separate entries.

## 4. Conclusions

This review synthesises the research on the carob tree over the last 40 years. The results reveal significant gaps in the scientific knowledge of this under-utilised crop species with fruit pulp and seeds of high industrial value [14]. These gaps include the biology of the species and its agronomic practices in comparison to other Mediterranean fruit trees such as almonds and olives [114]. The edibility of the fruit appears to be dependent on its industrial use rather than its inherent qualities. In the early 21st century, its commercial value was primarily linked to the smallest part of the fruit, the seed’s endosperm, but today, the whole fruit (pulp and seed) is highly valued for its health benefits [14,24,136]. Despite the industrial value of the fruit, it is important to continue researching the pharmacological validation of active pod constituents and their inclusion in clinical practice. This will increase the overall value of the fruit and diversify its uses, which is crucial for the economic value of the crop [122].

**Figure 1:**
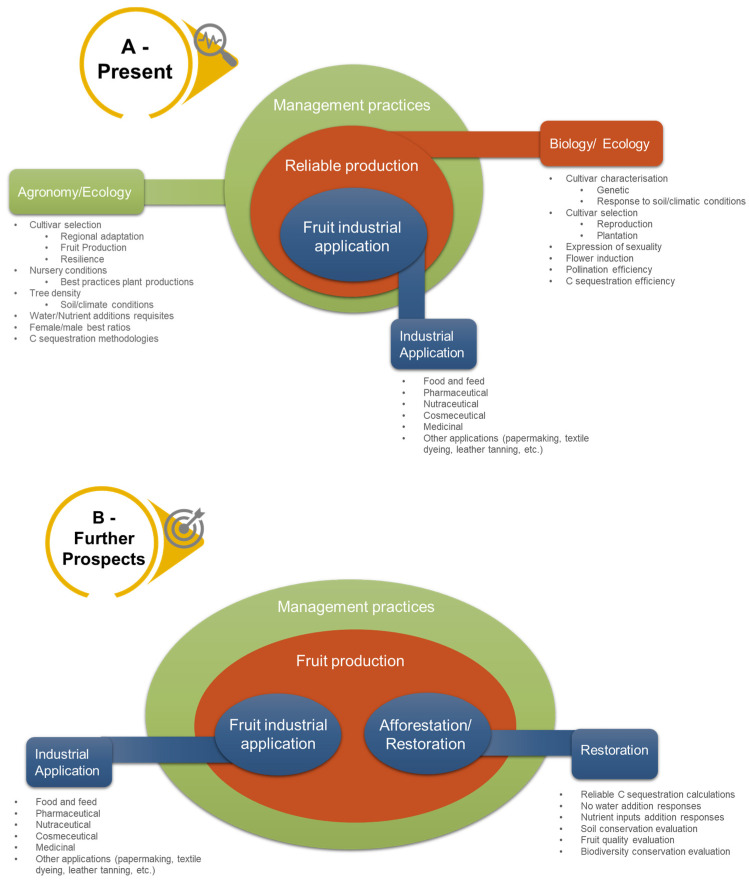
Outcome of hierarchical gaps in carob research knowledge that need to be addressed to ensure a reliable and resilient crop value. (**A**) The current value of the crop is dependent on basic and systematic knowledge of both the biology and the agronomy of the species. The different gaps for each part are listed. The nutrition and health industrial applications require further focused studies, particularly to gain better knowledge of possible uses and to conduct additional clinical trials. (**B**) Future prospects of carob use extend beyond industrial applications and include economic opportunities for farmers in arid and semi-arid regions. Under arid and semi-arid conditions, unsuitable for many other crops, carob trees can diversify farmers’ income streams, improve soil health, and mitigate the impacts of climate change through carbon sequestration and soil and biodiversity conservation measures.

The availability of carob pods on a transnational level is a major concern for the industry, and it is important that they are available throughout the year. This can be challenging if fruit production in the region declines or varies greatly between regions and years [8,109,123,136], which depends on both soil characteristics and climate. It was also discovered that mature carob orchards have irregularities in fruit production [116], which is a disadvantage for commercial purposes. Figure 1 displays the results of this bibliographic search, highlighting clusters with various knowledge gaps that require further investigation and investment.

Improving production is closely linked to implementing best management practices and investing capital. However, to determine the best management practices, it is necessary to have scientific knowledge of the varieties and their ecological and agronomic responses to different pedo-climatic conditions.

Basic scientific and agronomic research is needed to develop best management practices in carob orchards to make the carob tree a profitable resource for the future (Figure 1A,B). Moreover, the use of carob in reforestation or restoration programmes can provide a valuable crop while ensuring biodiversity conservation [120] and soil restoration. In addition to industrial applications, carob trees have been recognised as a new source of income due to their carbon sequestration potential [116,134] and as a biomonitor of air pollution [137]. This makes them a promising alternative to fight climate change and could increase the profitability of traditional crops in southern Europe in the long term (Figure 1B).

In times of environmental stress, carob cultivation can help farmers sustain their livestock. Carob trees are well adapted to arid and semi-arid climates and thrive with minimal water and soil fertility requirements. There is also untapped potential for developing novel carob-based products and applications. Although carob powder and gum are the most commonly used products, many other components of the carob tree could be explored for commercial use [14,24,136]. For instance, carob leaves contain tannins, which have applications in industries such as leather tanning, textile dyeing, and pharmaceuticals. Additionally, carob wood is dense and durable, making it suitable for construction, furniture making, and handicrafts. Carob waste can also be valued as a sugar platform that can be used as an alternative source of biofuels or even as an organic soil amendment [138,139,140].

As a whole, carob holds untapped value that presents opportunities for innovation, economic development, and environmental sustainability. By exploring the medicinal properties, environmental benefits, and commercial potential of carob, we can discover new avenues for its use and create a brighter future for this undervalued Mediterranean tree species. As the demand for healthy and environmentally friendly products continues to grow, the industrial value of the carob tree is likely to increase. This will drive innovation and investment in its cultivation and use. However, as we have pointed out, there are still many research gaps that need to be filled.

## Figures and Tables

**Table 1:** Distribution of articles on carob in the Scopus database over the last 40 years based on advanced searches.

Resource Uses	Screening Words	Specified	2000–2023	1980–2000
Selection of Cultivars	Cultivars	Characterisation	48	5
	Genetics		37	
Agronomic Production	Crop production		79	2
	Crop cultivation	Data	18	1
Industrial Applications			156	5
	Health	Effects	112	
		Benefits	44	
	Nutrition		158	12
Afforestation/Restoration	Farming		3	5
		Dry farming	4	
	Agroforestry		2	
	Environment	Ecological responses	77	20
